# Erythrocytes efficiently utilize exogenous sphingosines for S1P synthesis and export via Mfsd2b

**DOI:** 10.1074/jbc.RA120.012941

**Published:** 2021-01-23

**Authors:** Toan Q. Nguyen, Thiet Minh Vu, Farhana Tukijan, Sneha Muralidharan, Juat Chin Foo, Jasmine Fei Li Chin, Zafrul Hasan, Federico Torta, Long N. Nguyen

**Affiliations:** 1 Department of Biochemistry, Yong Loo Lin School of Medicine, National University of Singapore, Singapore; 2 Department of Medicine, Yong Loo Lin School of Medicine, National University of Singapore, Singapore; 3 Mechanobiology Institute, National University of Singapore, Singapore; 4 SLING/Immunology Program, Life Sciences Institute, National University of Singapore, Singapore; 5 Immunology Translational and Cardiovascular Disease Research Programme, Yong Loo Lin School of Medicine, National University of Singapore, Singapore

**Keywords:** sphingosine, sphingosine-1-phosphate, S1P transporter, Mfsd2b, BSA, bovine serum albumin, CCCP, carbonyl cyanide 3-chlorophenylhydrazone, CETSA, cellular thermal shift assay, MFS, Major Facilitator Superfamily, S1P, phingosine-1-phosphate, SphK, sphingosine kinases

## Abstract

Sphingosine-1-phosphate (S1P) is a potent lipid mediator that exerts its activity via activation of five different G protein–coupled receptors, designated as S1P1–5. This potent lipid mediator is synthesized from the sphingosine precursor by two sphingosine kinases (SphK1 and 2) and must be exported to exert extracellular signaling functions. We recently identified Mfsd2b as the S1P transporter in the hematopoietic system. However, the sources of sphingosine for S1P synthesis and the transport mechanism of Mfsd2b in erythrocytes remain to be determined. Here, we show that erythrocytes efficiently take up exogenous sphingosine and that a *de novo* synthesis pathway in part provides sphingosines to erythrocytes. The uptake of sphingosine in erythrocytes is facilitated by the activity of SphK1. By converting sphingosine into S1P, SphK1 indirectly increases the influx of sphingosine, a process that is irreversible in erythrocytes. Our results explain for the abnormally high amount of sphingosine accumulation in Mfsd2b knockout erythrocytes. Furthermore, we show that Mfsd2b utilizes a proton gradient to facilitate the release of S1P. The negatively charged residues D95 and T157 are essential for Mfsd2b transport activity. Of interest, we also discovered an S1P analog that inhibits S1P export from erythrocytes, providing evidence that sphingosine analogs can be used to inhibit S1P export by Mfsd2b. Collectively, our results highlight that erythrocytes are efficient in sphingosine uptake for S1P production and the release of S1P is dependent on Mfsd2b functions.

Sphingosine-1-phosphate (S1P), which is the phosphorylated product of sphingosine by sphingosine kinases 1 and 2 (SphK1 and 2) enzymes, is an abundant lipid mediator secreted by several cell types. The lipid mediator exerts its signaling activity by activating five different G protein–coupled receptors (S1P1–5) expressed in various cell types. Depending on the specific receptor subtype, S1P stimulates a myriad of cellular functions including cell mobility, proliferation, death, and differentiation in both an autocrine and a paracrine fashion. It is now established that S1P is required to sustain endothelial cell integrity and functions by increasing the expression of tight junction proteins. This signaling pathway also protects endothelial cells from inflammation by antagonizing the expression of adhesion molecules such as ICAM-1 and VCAM-1, which are the adhesion molecules for leukocytes rolling and transverse of blood vessels (1). S1P also regulates vascular tone and blood pressure (2, 3). In addition, the signaling is critical for lymphocyte trafficking and localization in lymphoid organs (4, 5, 6, 7). Targeting S1P signaling in lymphocytes has been utilized for treatment of multiple sclerosis (8). Thus, the S1P signaling pathway holds great promises for the treatment of various inflammatory conditions.

Sphingosine-1-phosphate is a zwitterionic lipid. Thus, it must be transported by transport proteins to function as an extracellular signaling molecule. One of the major challenges in the S1P signaling field is the identification of S1P transporters. ATP-dependent transporters such as ABCC1 and ABCC5 were identified as potential S1P transporters (9, 10). However, none of these ATP transporters has yet to be proven as physiological S1P transporters. The discovery of protein spinster homolog 2 (Spns2) as a first *bona fide* S1P transporter in the lymphatic system has partially explained for the sources of S1P (11). It is understood that Spns2 exports S1P from lymphatic endothelial cells and that it contributes a minor amount of plasma S1P (6, 12). However, it seems that Spns2 is not the major S1P transporter in blood cells (12, 13). Works from several groups have identified that erythrocytes contribute a large amount of plasma. However, the identification of this S1P transporter was only accomplished recently by our group. We show that Mfsd2b, which is highly expressed in erythrocytes and platelets, exports S1P (14, 15). Mfsd2b contributes approximately 50% plasma S1P. As the major cell type in blood, Mfsd2b-dependent erythrocytes is believed to contribute a major part. Nevertheless, the mechanism by which erythrocytes assimilate sphingosines for S1P synthesis and release is not completely understood.

Mfsd2b belongs to a group of solute carrier proteins termed Major Facilitator Superfamily (MFS) transporters. It is a homologous protein to Mfsd2a, the lysophosphatidylcholine transporter (16). Most of MFS transporters such as glucose transporters (Glut1–5), monocarboxylate transporters (MCT transporter family) transport water-soluble substrates. A few MFS transporters such as Spns2, Mfsd2a, and Mfsd2b transport lipid molecules. These lipid transporters may utilize a different mechanism to move lipophilic ligands through their cavity. In addition, some of the MFS transporters utilize cations to drive the transport of ligands (17). Here, we investigate the mechanisms by which erythrocytes generate and release the S1P via Mfsd2b. We found that erythrocytes efficiently take up sphingosine for S1P synthesis. The influx of sphingosine is regulated by sphingosine kinase 1 (SphK1). Furthermore, a gradient of proton is necessary to enhance S1P release activity in erythrocytes. Finally, we discover an S1P analog that may function as a competitive inhibitor of Mfsd2b.

## Results

### Erythrocytes take up exogenous sphingosines for S1P synthesis and release in an Mfsd2b-dependent manner

**Figure 1:**
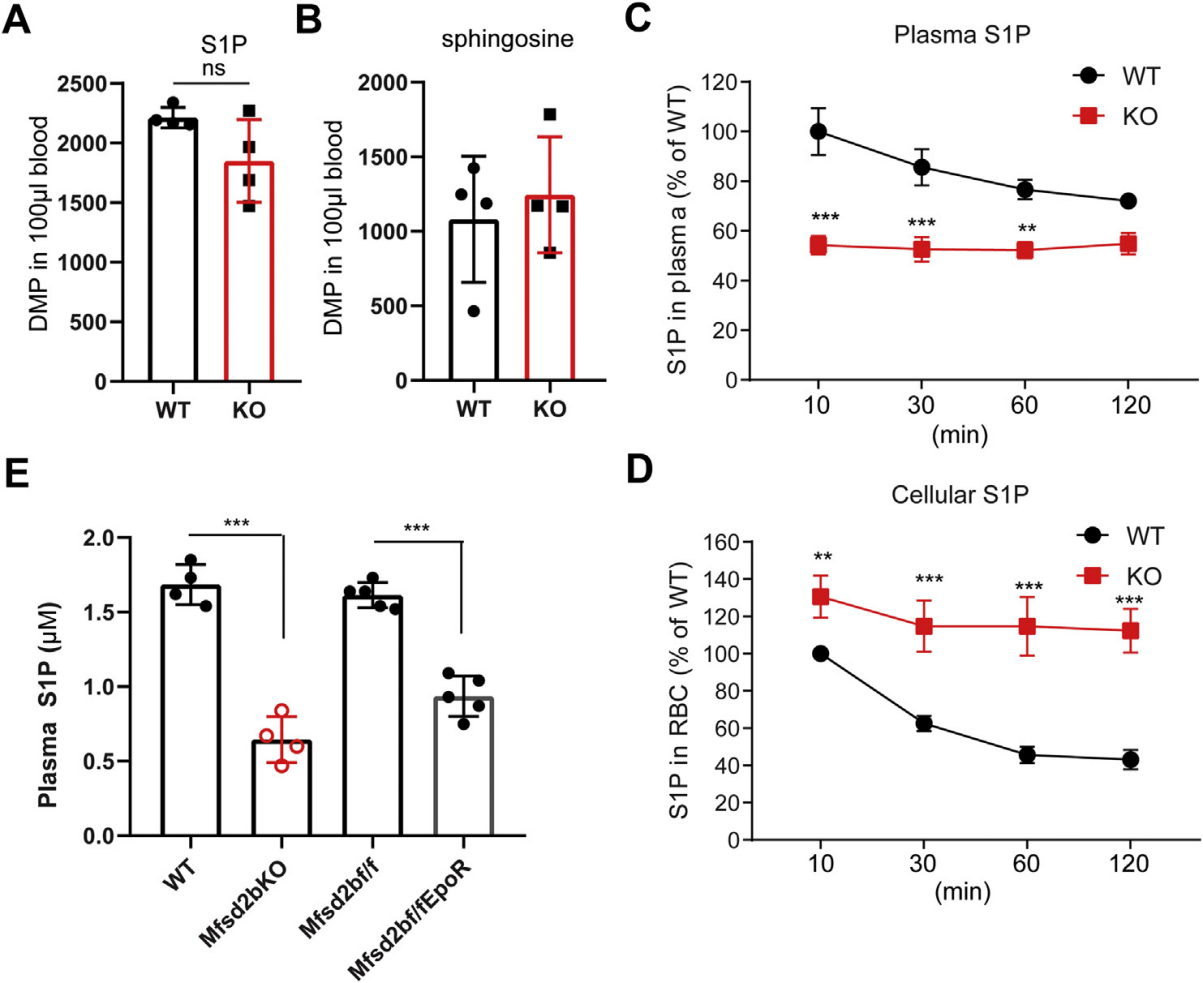
**Erythrocytes efficiently import sphingosines for S1P synthesis and release**. *A*, *B*, radioactive signals of S1P and sphingosine isolated from whole blood (blood cells and plasma) at 1 min after i.v. injection with radioactive sphingosine. There is no significant difference in the amount of S1P produced and sphingosine leftover in whole blood samples between WT and KO at 1 min. Experiments were performed twice with n = 4 to 6 mice for each genotype. *C*, *D*, time course of S1P production in plasma and blood cells after IV injection with sphingosine. We noted that exogenous sphingosine was quickly taken up for S1P synthesis and released by red blood cells. The highest level of S1P was detected between 1 and 10 min after sphingosine injection and reduced over the indicated times in WT mice. Intracellular S1P levels from blood cells were also reduced over time, indicative of active export of S1P. Owing to technical limitations, we only collected samples from 10 min onward. Experiments were performed twice with n = 7 to 8 mice per genotype. ∗∗*p* < 0.01, ∗∗∗*p* < 0.001; *p* values were calculated using two-way ANOVA. *E*, lipidomics analysis of plasma S1P from WT, global KO, Mfsd2bf/f, and Mfsd2bf/fEpoR-Cre mice. Data are mean and SD, n = 4 to 5 per genotypes, ∗∗∗*p* < 0.001. *p* Values were calculated using one-way ANOVA. ns, not significant.

Incubation of isolated erythrocytes with exogenous sphingosine led to intracellular synthesis of S1P, which is then released via Mfsd2b (14), indicating that exogenous sphingosines can be used for S1P synthesis. However, it is unclear whether *in vivo* erythrocytes also take up sphingosines for S1P synthesis. Thus, we intravenously injected with a bolus of radioactive [3-^3^H]-sphingosine solubilized in bovine serum albumin (BSA) into WT and global Mfsd2b knockout (KO) mice and measured [3-^3^H]-S1P in plasma and erythrocytes. We found that exogenous sphingosine is quickly taken up by both WT and KO erythrocytes and used for S1P synthesis (Fig. 1, *A*–*B*). After 10-min injection, plasma S1P levels in WT mice were significantly greater than that of KO mice (Fig. 1*C*). However, the plasma S1P level in WT was not increased over time, instead it was slightly decreased (Fig. 1*C*). In addition, the intracellular level of S1P in WT erythrocytes mice was also significantly decreased over time (Fig. 1*D*). These results suggest that a bolus injection of sphingosine was utilized by blood cells and that WT erythrocytes release S1P into the circulation that is quickly turned over, in line with a short half-life of S1P (18). In comparison with WT mice, plasma S1P levels in KO mice remained constantly about 50% of WT S1P levels. In addition, KO erythrocytes accumulated with significantly higher levels of S1P (Fig. 1, *C*–*D*). These results indicate that erythrocytes efficiently assimilate exogenous sphingosine for S1P synthesis and release via Mfsd2b.

Several proteins including Spns2 and ABC transporters have been shown to have S1P transport functions. We showed that erythrocytes do not express Spns2 (Fig. S1*A*), while Mfsd2b is present as previously shown (14). In addition, we performed S1P transport assays using erythrocytes isolated from global Mfsd2b KO and Spns2 KO mice. Consistently with previous findings (13), erythrocytes isolated from global Spns2 KO exhibit normal S1P transport activity that is comparable with that of WT cells, whereas Mfsd2b KO erythrocytes showed a significant reduction of S1P transport (Fig. S1, *B*–*C*). These findings indicate that Mfsd2b, but not Spns2, is the S1P transporter in erythrocytes, explaining the remarkably high level of S1P accumulation in KO erythrocytes as shown previously by our results (14). Mfsd2b-expressing cells including erythrocytes and platelets contribute approximately 50% of the total S1P in plasma (14). We generated erythrocyte-specific knockout of Mfsd2b (referred to as Mfsd2bf/fEpoR-Cre). Analysis of plasma S1P levels from Mfsd2bf/fEpoR-Cre, global Mfsd2b KO mice, and their respective controls showed that S1P levels in Mfsd2bf/fEpoR-Cre mice and global Mfsd2b KO mice had approximately 50% levels of control mice (Fig. 1*E*). Taken together, our results indicate that erythrocytes provide a major pool of plasma S1P via Mfsd2b.

### Sphingosine kinase 1 facilitates the uptake of exogenous sphingosine in erythrocytes

**Figure 2:**
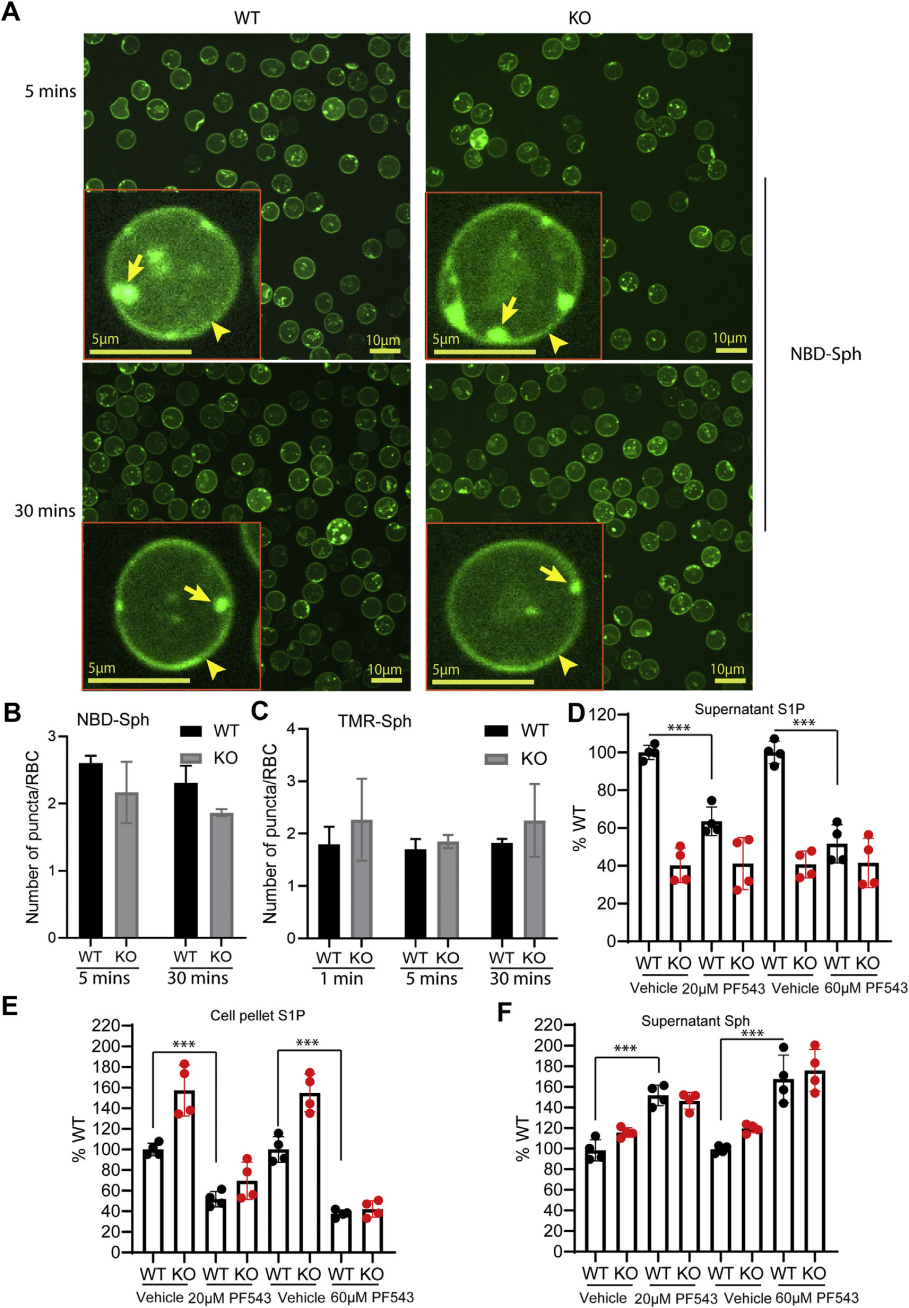
**Inhibition of SphK1 reduces sphingosine uptake in erythrocytes**. *A*, distribution of NBD-S1P in WT and KO red blood cells after incubation of NBD-sphingosine for 5 and 30 min. We interpreted the green signals as NBD-S1P as we have observed that the NBD-Sph level is low in red blood cells (see Results in Vu *et al.* [14]). However, we cannot discriminate NBD-S1P and NBD-Sph signals by microscopic assays. Representative images from three WT and three KO mice. Similar results were obtained for TMR-Sph. *Arrowheads* and *arrows* show NBD-S1P signals on the plasma membrane and in the cytoplasm, respectively. *B*, *C*, quantification of numbers of puncta in WT and Mfsd2b KO erythrocytes after incubation with NBD-Sph or TMR-Sph, respectively. n = 3 for each genotype. *D*, *E*, S1P levels in supernatant and cell pellets with or without treatment of 20 and 60 μM PF543. Radioactive S1P was isolated from the supernatant and cell pellets for quantification. S1P signals from WT without treatment were set as 100%. *F*, Sphingosine levels in the supernatant with or without 20 and 60 μM PF543. In these experiments, 2 μM [3-^3^H]-Sph was used in “continuous” assays for 15 min. Sphingosine signals from WT without treatment were set as 100%. Data are mean and SD, n = 4 per genotype. Experiments were repeated twice. ∗∗∗*p* < 0.001. *p* Values were calculated using one-way ANOVA.

To gain more insight into how erythrocytes take up exogenous sphingosines, we utilized a fluorescent-labeled sphingosine (NBD-Sph) to observe its transport routes in red blood cells (RBCs). Upon incubation of NBD-Sph with WT or Mfsd2b KO RBC, we found that the majority of the fluorescent signals was localized in the plasma membrane (Fig. 2*A*, arrowheads). Of note, we also found several small NBD-labeled puncta present in the cytoplasm and the plasma membrane of WT and Mfsd2b KO RBCs (Fig. 2*A*, arrows). There were a few puncta present in each erythrocyte, and the number of puncta was not significantly different between WT and KO erythrocytes (Fig. 2*B*). This phenotype was also observed with another sphingosine analog, TMR-Sph (Fig. 2*C* and Fig. S2). The nature of these NBD-labeled puncta is unclear, but they were negative for Ter119, a plasma membrane marker for RBC (Fig. S3), ruling out the involvement of endocytosis as a route for sphingosine transport in RBC.

Deletion of SphK1 in erythrocytes caused an approximately twofold increase of sphingosine (19), whereas deletion of Mfsd2b resulted in an approximately tenfold increase of sphingosines in erythrocytes (14). These data suggest that conversion of sphingosine into S1P facilitated by SphK1 is necessary to drive the influx of sphingosine in erythrocytes. Thus, we tested whether inhibition of SphK1 with PF543, a specific inhibitor for SphK1, could reduce the uptake of sphingosine (3). Indeed, PF543 treatment resulted in a significant reduction of extracellular and intracellular S1P in WT and Mfsd2b KO cells, indicating that PF543 inhibits S1P synthesis and reduces total S1P levels (Fig. 2, *D*–*E*). We found that inhibition of SphK1 with PF543 significantly reduced sphingosine uptake in both WT and Mfsd2b KO RBCs as there was a significantly higher amount of sphingosine present in the medium (Fig. 2*F*). Mfsd2b deletion did not affect sphingosine uptake (Fig. 2*F*). Together, our results indicate that phosphorylation of sphingosine by SphK1 facilitates its influx into erythrocytes. These results also indicate that Mfsd2b works downstream of SphK1 pathway.

### *De novo* synthesis of sphingolipids in part provides sphingosines for S1P synthesis in erythrocytes

**Figure 3:**
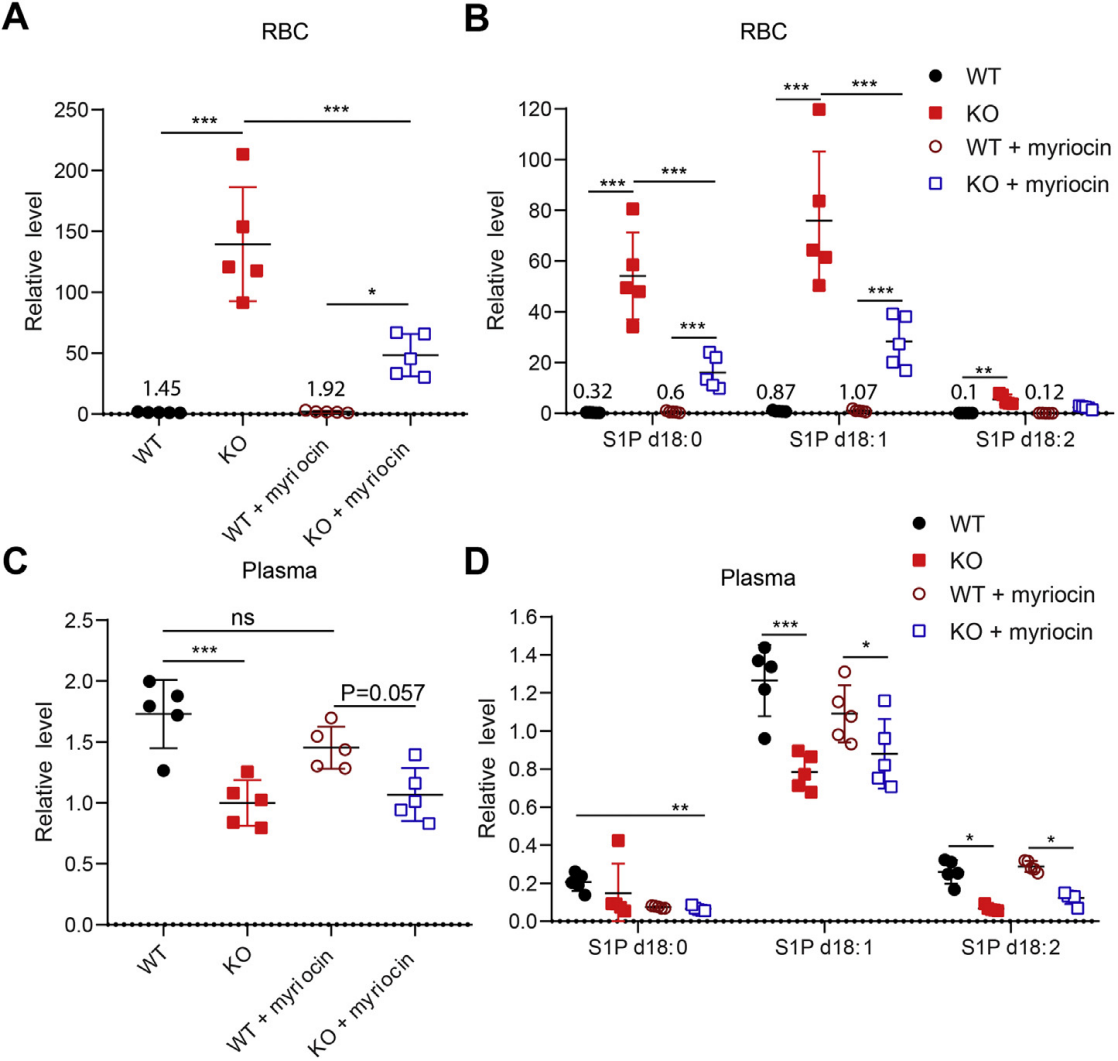
***De novo* sphingolipid synthesis generates sphingosines for S1P synthesis in erythrocytes.***A*, *B*, total and individual S1P species levels in WT and Mfsd2b KO erythrocytes before and after 24 days treatment with myriocin. Numbers show the average levels of S1P in WT. *C*, *D*, total and individual S1P species levels in WT and Mfsd2b KO plasma treated or untreated with myriocin for 24 days. Erythrocytes and plasma were collected for lipidomic analysis. Data are mean and SD, n = 5 per genotypes. ∗*p* < 0.05, ∗∗*p* < 0.01, ∗∗∗*p* < 0.001, ns, not significant. *p* Values were calculated using one-way ANOVA.

The range of plasma concentration of sphingosines including dihydrosphingosine (dhSph) and sphingosine is from 50 to 150 nM (14, 20). Our results above suggest that sphingosine in plasma can be used by the SphK1-Mfsd2b axis in erythrocytes for S1P synthesis and release. To gain more insight into the source of sphingosines for S1P synthesis *in vivo*, we utilized myriocin, a specific inhibitor of the rate-limiting enzyme serine palmitoyltransferase, SPT1 (also known as Sptlc1) to inhibit *de novo* synthesis of sphingolipids. Wildtype and Mfsd2b KO mice were treated with myriocin for 24 days (21). Erythrocytes and plasma samples were collected for S1P analysis. In erythrocytes, we found that the total S1P levels in Mfsd2b KO RBC were significantly reduced in myriocin-treated conditions, indicating that *de novo* synthesis of sphingolipids is involved in providing sphingosines for S1P synthesis (Fig. 3*A*). Myriocin directly inhibits the synthesis of dihydrosphingosine (dhSph), which is subsequently used for dihydrosphingosine-1-phosphate (S1P d18:0). In addition, we found that myriocin treatment also decreased S1P (d18:1) and S1P (d18:2) levels in Mfsd2b KO RBC (Fig. 3*B*). These results suggest that *de novo* synthesis of sphingolipids is also indirectly involved in providing the precursors for the synthesis of these S1P species (22). Although S1P species levels were significantly decreased after myriocin treatment, Mfsd2b KO RBC still had a significantly higher level of S1P compared with WT cells, suggesting that other pathways may contribute to the sources of sphingosine (Fig. 3*B*). Thus, we examined whether a hydrolysis of endogenous sphingomyelin or *de novo* synthesis of sphingolipids in erythrocytes would provide additional sources of sphingosine for S1P synthesis. Treatment of washed RBC with amitriptyline or myriocin did not reduce the endogenous S1P level, indicating that hydrolysis of SM and *de novo* synthesis of sphingosines are not active in RBC (Fig. S4*A*). The sphingomyelinase activity from WT and Mfsd2b KO RBCs was not detectable, suggesting that the hydrolysis of sphingolipids is not active in RBC (Fig. S4*B*). Our data suggest, perhaps, myriocin treatment was incomplete. In plasma, we did not observe a significant decrease in the total level of S1P after myriocin treatment (Fig. 3*C*), with the exception that dihydrosphingosine-1-phosphate was significantly reduced after myriocin treatment in WT mice (Fig. 3*D*). Together, these results indicate that *de novo* synthesis of the sphingolipid pathway, rather than endogenous sources, partially provides substrates for S1P generation in erythrocytes.

### A proton gradient increases S1P export activity in erythrocytes

**Figure 4:**
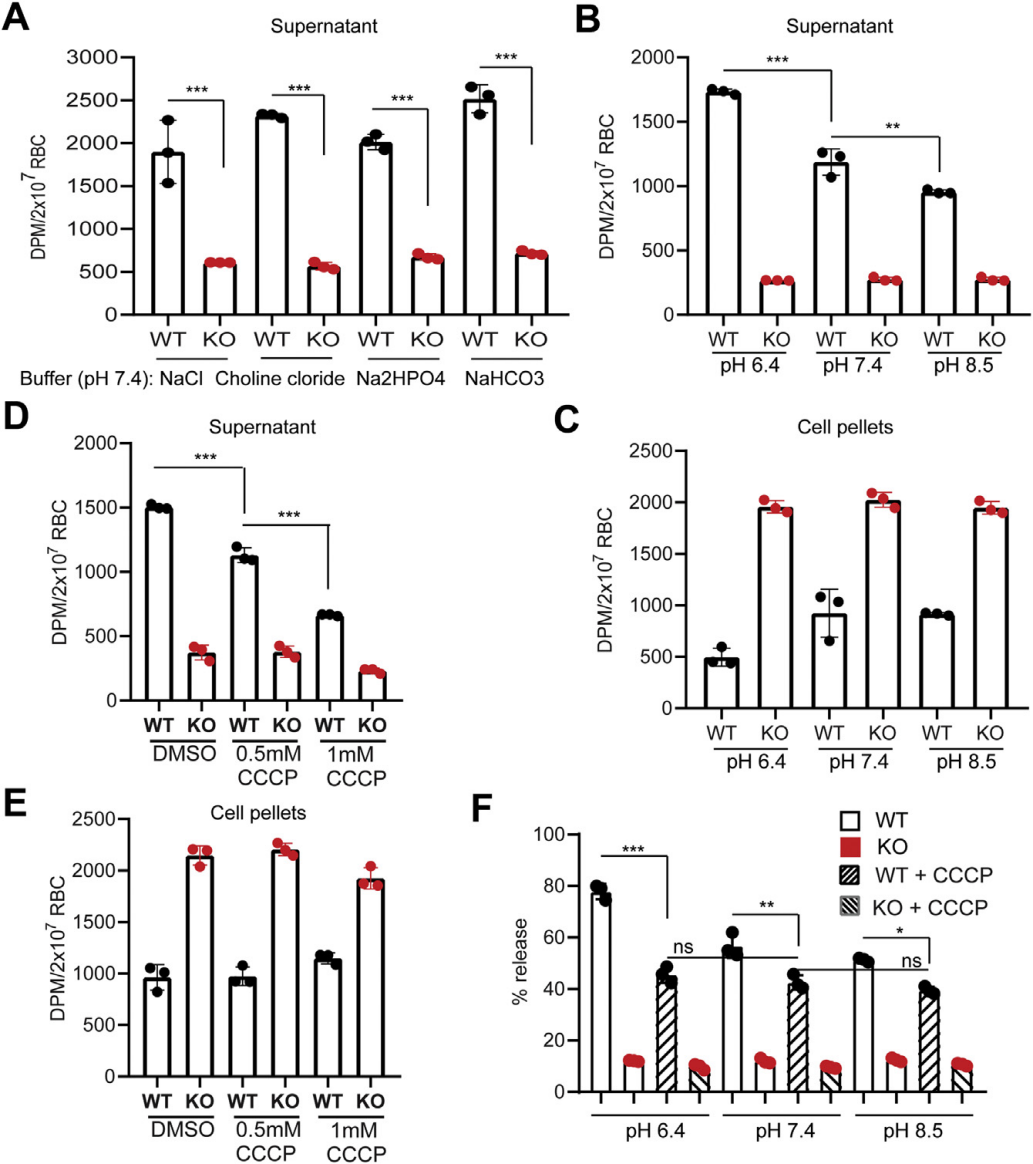
**Proton gradient affects S1P export activity in RBC**. *A*, transport activity of S1P in WT and Mfsd2b KO red blood cells (RBCs) at indicated buffers. Removal of sodium or chloride does not affect S1P export activity. *B*, *C*, effects of pH levels on S1P release from WT RBC. We noted that S1P synthesis in WT and KO RBCs in the pHs was unchanged. A lower pH increases and higher pH decreases the release of S1P. *D*, *E*, effects of CCCP (carbonyl cyanide 3-chlorophenylhydrazone) on S1P release from WT and KO RBCs at pH 7.4. *F*, effects of CCCP on S1P release from WT and KO RBCs at indicated pHs. In these experiments, wildtype and KO RBCs were preloaded with S1P in corresponding pHs in “preloading” assays. S1P release was stimulated by the addition of 0.5% bovine serum albumin. We noted that 0.5% bovine serum albumin used to capture S1P release in this assay strongly reduces the effect of CCCP. Therefore, we only observed the effects of CCCP with indicated concentrations. Data are expressed as mean and SD. n = 3 per genotype. Experiments were repeated twice. ∗*p* < 0.05, ∗∗*p* < 0.01, ∗∗∗*p* < 0.001. *p* Values were calculated using one-way ANOVA.

In erythrocytes, Band3 (also called Slc4a1) is a highly abundant protein, which is critical to maintain membrane potential by exchanging anions. It is intriguing that, inhibition of Band3 has been reported to affect S1P release in erythrocytes (23). In addition, glyburide, an inhibitor of ATP-sensitive potassium channel and several proton pump inhibitors were shown to affect S1P release from erythrocytes (Fig. S5) (10, 24). These results suggest that S1P synthesis and release in erythrocytes are sensitive to changes in proton concentrations. To test whether Mfsd2b utilizes sodium to drive S1P transport, we replaced sodium with choline in the transport buffer. However, replacement of sodium with choline did not result in the reduction of S1P export in RBC (Fig. 4*A*), indicating that sodium gradient is not important to generate energy for S1P export. We also ruled out the requirements of other cations and anions for the S1P transport by Mfsd2b (Fig. 4*A*). Of interest, Mfsd2b activity was significantly more active at pH 6.4 than at physiological pH 7.4 and became less active at pH 8.5 (Fig. 4, *B*–*C*). These data implicate that a gradient of protons is necessary for the increased release of S1P. Proton ionophores cause leak of protons and disrupt the membrane potential. Treatment of WT and KO erythrocytes with carbonyl cyanide 3-chlorophenylhydrazone (CCCP) significantly reduced S1P export at pH 7.4 (Fig. 4, *D*–*E*). We showed that CCCP reduced S1P transport activity facilitated by lower pH (Fig. 4*F*). However, CCCP did not completely abolish S1P release in WT cells (Fig. 4*F*). Collectively, our results strongly suggest that a gradient of proton is necessary to increase S1P export activity of Mfsd2b in erythrocytes.

### Possible involvement of D95 and T157 in Mfsd2b for proton binding

**Figure 5:**
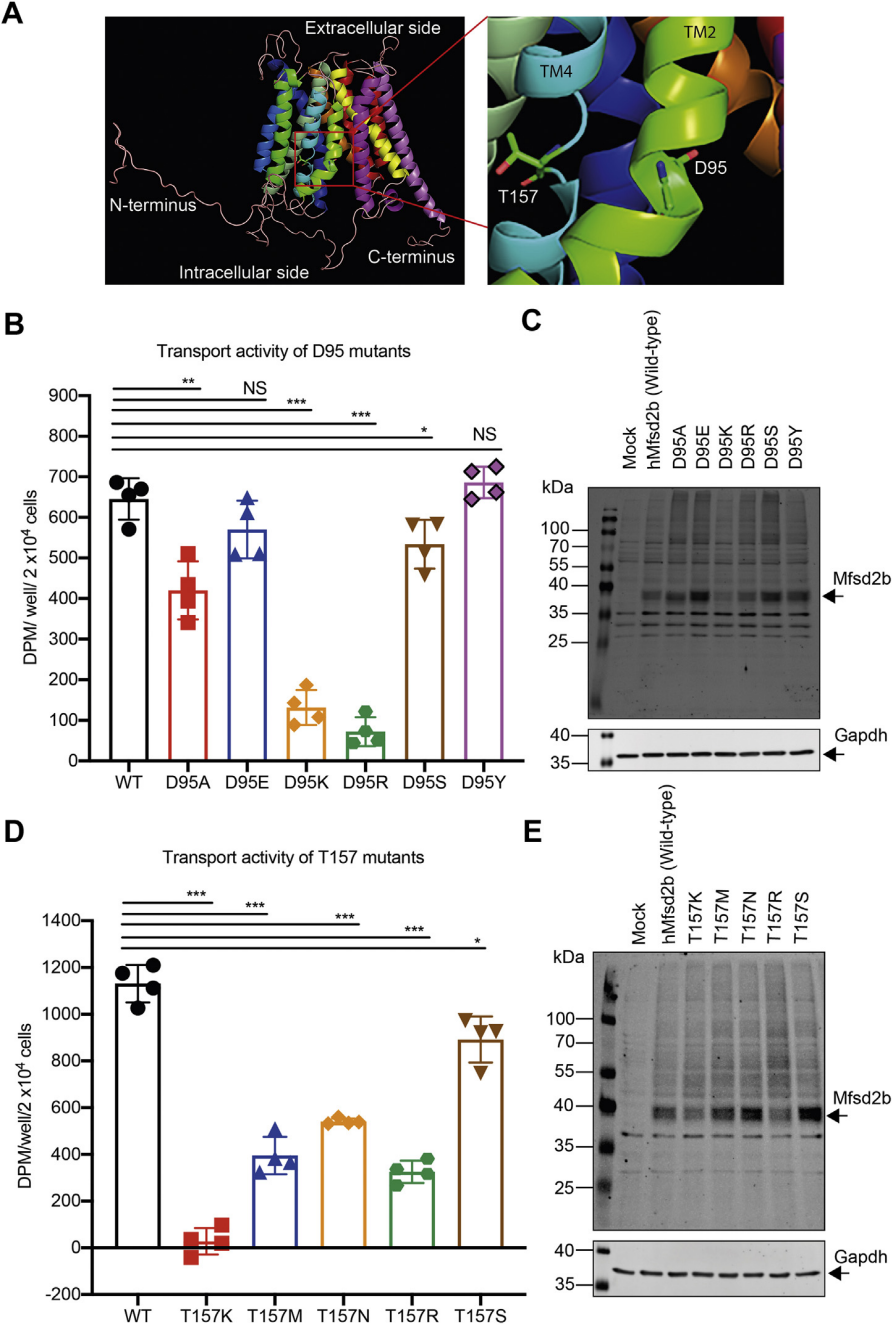
**Aspartate 95 and threonine 157 residues are required for transport activity**. *A*, modeled structure of human Mfsd2b. Shown are the locations of D95 and T195 residues on transmembrane (TM) 2 and 4, respectively. *B*, transport activity of mock, wildtype Mfsd2b (WT), D95A, D95E, D95K, D95R, D95S, and D95Y mutants. Transport activity of D95A, D95K, and D95R was significantly reduced, whereas the transport activity of D95E, D95S, and D95Y was almost rescued to that of WT activity. These data suggested that the carboxyl group from D95 may be involved in the interaction with proton during S1P transport. *C*, Western blot analysis of wildtype Mfsd2b (WT), D95A, D95E, D95K, D95R, D95S, and D95Y mutants. Expression of these mutant proteins are similar to that of the wildtype protein. *D*, transport activity of mock, wildtype Mfsd2b (WT), T157M, T157N, T157R, T157K, and T157S mutants. Transport activity of T157M, T157N, T157R, T157K, and T157R was significantly reduced, whereas the transport activity of T157S was slightly reduced to that of WT activity. The data suggest that the hydroxyl group of T157 may be involved in the interaction with proton. *E*, Western blot analysis of wildtype Mfsd2b (WT), T157M, T157N, T157R, T157K, and T157S mutants. All of the T157 mutants have a normal protein expression pattern. Shown are radioactive signals from indicated conditions after subtraction to the signal from mock. Data are means and SD, ∗*p* < 0.05, ∗∗*p* < 0.01, ∗∗∗*p* < 0.001. ns, not significant. *p* Value was calculated using one-way ANOVA.

To gain more insight into the possible binding of proton to Mfsd2b residues, we investigated on the transport activity of D95 and T157 residues (Fig. 5*A*), which were predicted to be conserved residues for binding to sodium in Mfsd2a (25). For D95 residue, its substitution with alanine (D95A) resulted in the reduction of transport activity (14). Similarly, substitutions of D95 to lysine (D95K) and arginine (D95R) resulted in a significantly reduced transport activity without changing protein expression levels (Fig. 5, *B*–*C*). Of interest, the mutants D95S and D95Y exhibited strong transport activity without change in expression levels (Fig. 5, *B*–*C*). These results indicate that the carboxyl group from D95 is involved in the interaction with proton to maintain the transport activity of Mfsd2b (Fig. 5, *B*–*C*). Similarly, we also showed that a substitution of T157 with lysine (T157K), methionine (T157M), asparagine (T157N), or arginine (T157R) caused a significantly reduced transport activity in Mfsd2b (Fig. 5, *D*–*E*). The transport activity of T157S was comparable with that of the native protein, implying that the hydroxyl group from T157 is also important for Mfsd2b activity (Fig. 5, *D*–*E*). These results show that the residues D95 and T175 are likely involved in the interaction with proton and a gradient of proton is necessary to increase S1P export activity of Mfsd2b.

### Mfsd2b can release NBD-S1P but not TMR-S1P

**Figure 6:**
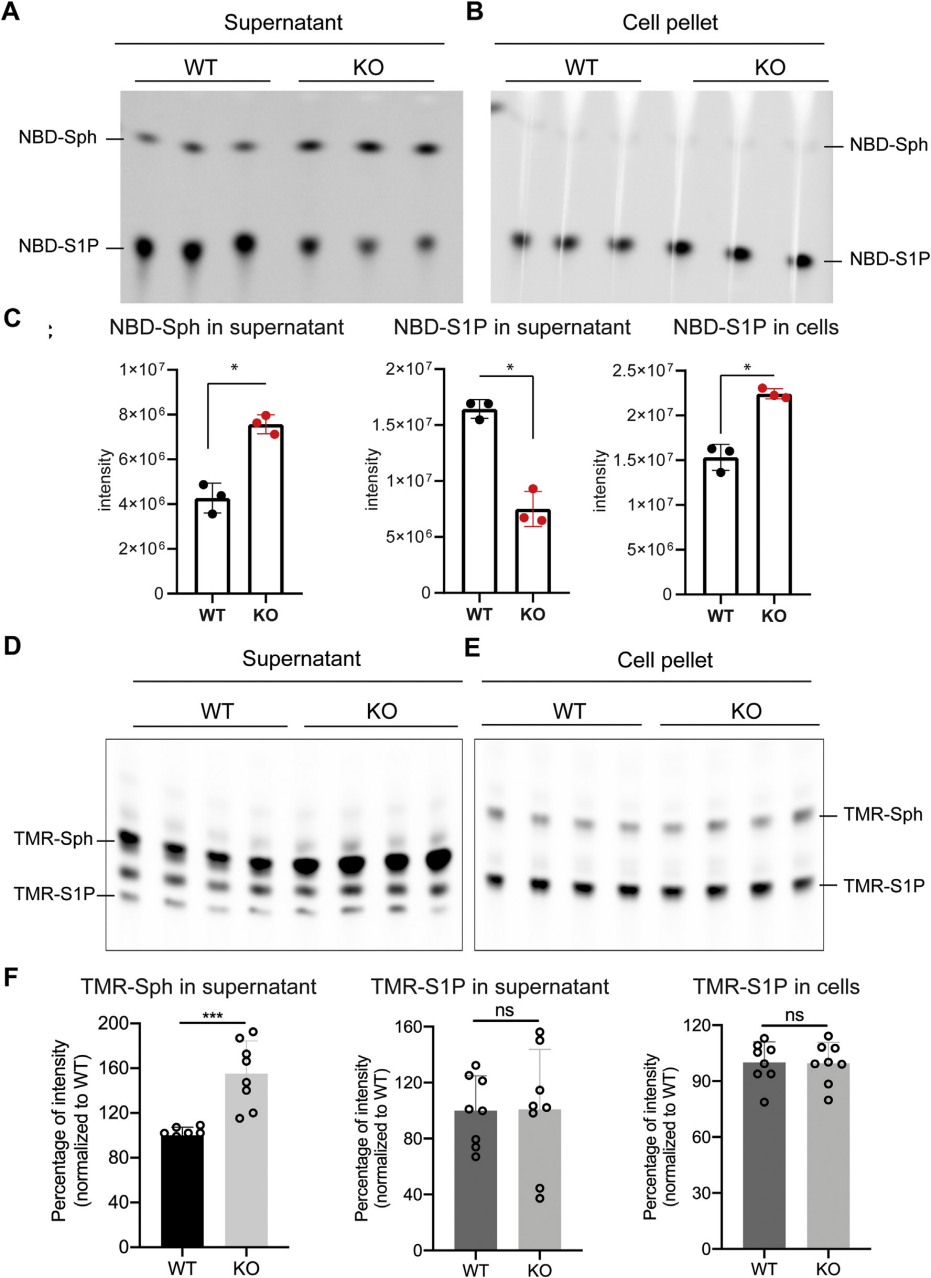
**Erythrocytes export NBD-S1P, but not TMR-S1P, in an Mfsd2b-dependent manner**. *A*, *B*, TLC analysis of WT and Mfsd2b KO erythrocytes after incubation with 2.5 μM NBD-sphingosine in “continuous” assays. *C*, quantification of NBD-Sph and NBD-S1P bands from A and B. n = 3 for each genotype. *D*, *E*, TLC analysis of WT and Mfsd2b KO erythrocytes after incubation for 1 h with 2.5 μM TMR-sphingosine in “continuous” assays. *F*, quantification of TMR-Sph and TMR-S1P bands from D and E. In these experiments, NBD-sphingosine and TMR-sphingosine were used as the substrates for synthesis of the S1P analogs. Each dot represents one animal. Data are means and SD, ∗*p* < 0.05, ∗∗*p* < 0.001. *p* Value was calculated using unpaired i-test. ns, not significant.

Mfsd2b belongs to the MFS transporters, which transport ligands through conformational changes. The alternative switching of outward and inward conformations facilitates the release of ligands through their transport cavity (26). Most of MFS transporters accept small molecules as ligands (17). Therefore, we tested the transport capability of Mfsd2b with sphingosine analogs, which are commercially available (Fig. 6). Except for fluorescein-sphingosine, we showed that NBD-sphingosine and TMR-sphingosine can be efficiently taken up by erythrocytes for synthesis of NBD-S1P and TMR-S1P, respectively (Fig. 6, *A*–*F* and Figs. S2 and S6). In agreement with published data (27), NBD-S1P can be released by WT erythrocytes, resulting in a lower level of intracellular NBD-S1P compared with Mfsd2b KO cells (Fig. 6, *A*–*C*). The results indicate that the NBD group can be transported through the Mfsd2b cavity. Unexpectedly, we found that release of TMR-S1P was not different between WT and KO RBCs, resulting in a simultaneous accumulation (Fig. 6, *D*–*F*). The uptake of TMR-Sph (and also NBD-Sph) was slightly reduced in Mfsd2b KO RBCs (Fig. 6*F*). These findings indicate that Mfsd2b is unable to export TMR-S1P.

### Inhibitory properties of TMR-S1P to the release of S1P in erythrocytes

**Figure 7:**
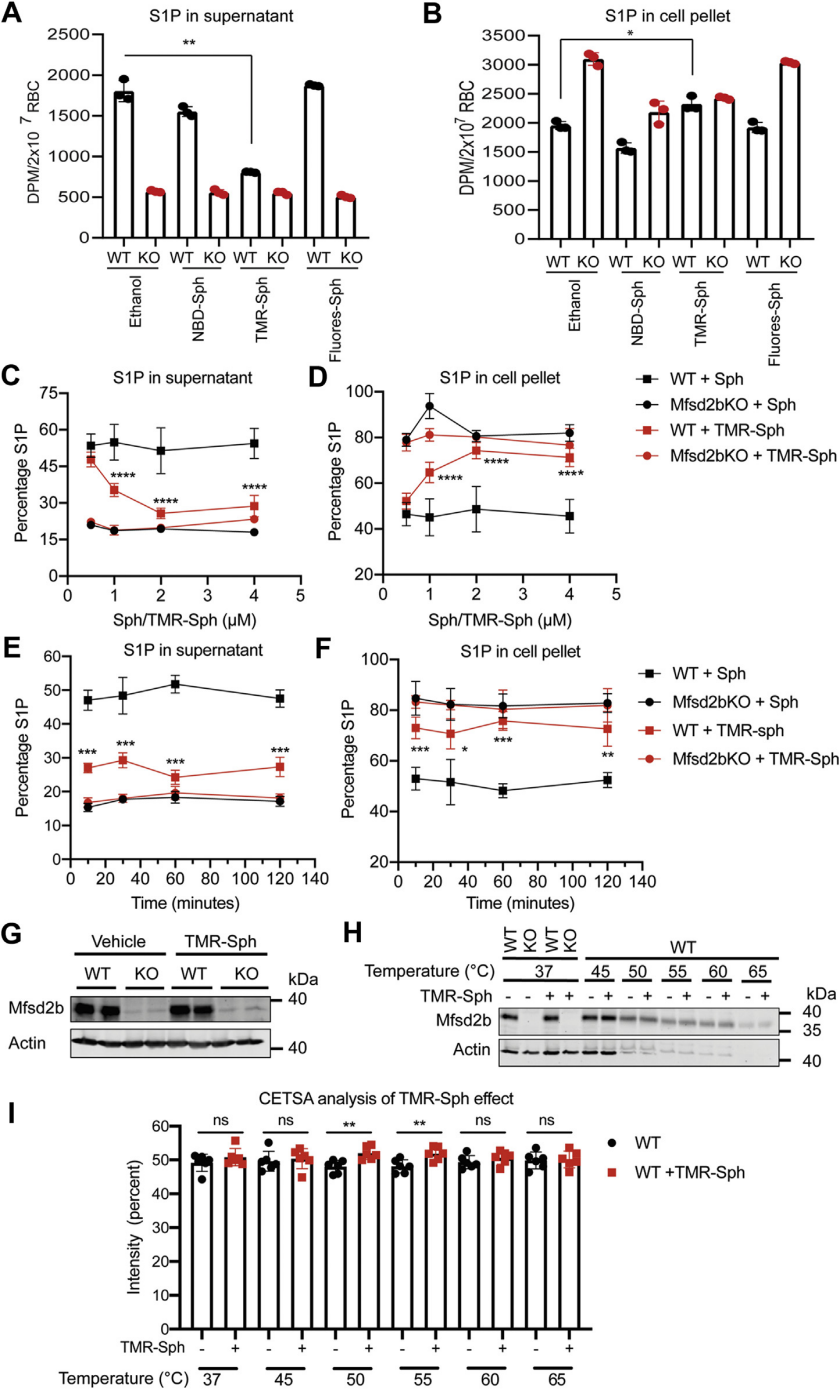
**TMR-S1P exhibits inhibitory properties to S1P release in erythrocytes**. *A*, *B*, competition assays of sphingosine analogs with [3-^3^H]-Sph. TMR-Sph treatment showed an inhibitory effect on [3-^3^H]-S1P release in WT red blood cells (RBCs) (*A*), which concomitantly increased intracellular levels of S1P (*B*). WT and Mfsd2b KO RBCs were preincubated with 2 μM indicated sphingosine analogs for 2 h, then 2 μM [3-^3^H]-Sph and 0.5% BSA were added. The mixture was further incubated for 40 min. *C*, *D*, dose-dependent inhibition of [3-^3^H]-S1P release from TMR-Sph treatment. TMR-Sph treatment reduced the extracellular level of [3-^3^H]-S1P (*C*), which increased the intracellular [3-^3^H]-S1P (*D*) from WT RBC. WT and KO RBCs were preincubated with an indicated concentration of either unlabeled sphingosine or TMR-Sph for 2 h, then 1 μM [3-^3^H]-Sph and 0.5% bovine serum albumin were added. The mixture was further incubated for 30 min. Ethanol was used as vehicle as sphingosine was dissolved in ethanal. In comparison with unlabeled sphingosine, TMR-Sph shows inhibitory effects on the release of [3-^3^H]-S1P. n = 4 for each genotype. *E*, *F*, time course of TMR-Sph treatment. WT and KO RBCs were preincubated with 2 μM TMR-Sph for the indicated times. Then 1 μM [3-^3^H]-Sph and 0.5% bovine serum albumin were added. The mixture was further incubated for 30 min before S1P from cell pellets and supernatant were isolated and quantified. n = 4 for each genotype. *G*, TMR-Sph treatment does not reduce expression of Mfsd2b in erythrocytes. *H*, *I*, cellular thermal shift assay (CETSA) of wildtype erythrocytes with TMR-S1P. Erythrocytes were treated with 2 μM TMR-Sph or ethanol as vehicle for 1 h. Treated erythrocytes were heated at indicated temperatures. Mfsd2b protein expression was analyzed by Western blot and quantified. Mfsd2b protein from erythrocytes treated with TMR-Sph was significantly more stable at 55 and 60 °C. n = 6 for each genotype. ∗*p* < 0.05, ∗∗*p* < 0.01, ∗∗∗*p* < 0.001. *p* Values were calculated using one-way ANOVA. Data are expressed as mean and SD.

Given the structural similarity between S1P and TMR-S1P, and the inability to be exported by Mfsd2b, we explored a possibility that TMR-S1P would compete with S1P for release via Mfsd2b. We utilized competition assays, in which [3-^3^H]-Sph was used as the substrate for S1P synthesis and TMR-Sph, NBD-Sph, fluorescein-Sph were used as cold competitors. We found that, after incubation with equal molar amounts of the competitors, only TMR-Sph exhibited inhibitory effects on [3-^3^H]-S1P release (Fig. 7*A*). As a result, the intracellular S1P level was concomitantly increased in TMR-Sph-treated cells compared with NBD-Sph and fluorescein-Sph, indicating that TMR-S1P possesses inhibitory effects on S1P export (Fig. 7*B*). NBD-Sph and fluorescein-Sph were unable to inhibit S1P release (Fig. 7*A*). To gain more insights into the inhibitory properties of TMR-S1P, we performed a dose-dependent assay. In these experiments, unlabeled sphingosine was used in parallel as control. Higher concentrations of TMR-Sph enhanced the inhibition of S1P release from erythrocytes (Fig. 7*C*). Concomitantly, TMR-S1P inhibition of S1P release caused significant accumulation of intracellular S1P levels in WT cells (Fig. 7*D*). TMR-Sph was quickly taken up by erythrocytes for inhibition of S1P release over time (Fig. 7, *E*–*F*). Treatment with TMR-Sph did not alter Mfsd2b expression in RBCs (Fig. 7*G*). These results strongly suggest that TMR-Sph is converted into TMR-S1P, which exhibits inhibitory effects to S1P release via Mfsd2b.

To gain further insights into a possible interaction of TMR-S1P with Mfsd2b, we used cellular thermal shift assay (CETSA). The direct interaction of TMR-S1P would stabilize Mfsd2b protein under heat treatment. Indeed, our data showed that treatment of TMR-Sph significantly increased the stability of Mfsd2b protein at 50 and 55 °C compared with controls, pointing to a possibly direct interaction of TMR-S1P to Mfsd2b (Fig. 7, *H*–*I*). Together, our findings show that TMR-S1P can inhibit S1P release in erythrocytes, possibly by competing with S1P for export via Mfsd2b.

## Discussion

Sphingosine-1-phosphate is a potent lipid mediator, which has gained much attention over the last two decades owing to its pleiotrophic effects on various cell types and physiological conditions. In mammalian cells, S1P is synthesized by SphK1 and 2, which are expressed ubiquitously. In most cell types, S1P is reversibly hydrolyzed by S1P phosphatases into sphingosines or irreversibly degraded by the S1P lyase, Sgpl1 (28). The by-products of S1P breakdown can be recycled for sphingosine and phospholipid synthesis (28). In cell types lacking these enzymes, S1P is released into the extracellular environment for signaling *via* five subtype receptors S1P1–5 (29). It has been documented that endothelial and blood cells simultaneously release S1P into the circulation (18, 30). The recent discoveries of S1P transporters have greatly advanced our understanding toward the sources and the physiological roles of S1P (11, 14). Our own group recently cloned Mfsd2b and showed that it is an S1P exporter in blood cells (14). Using knockout mice, we demonstrated that Mfsd2b expression in erythrocytes and platelets are a major contributor for plasma S1P. The current study aimed to provide further understandings about the mechanisms by which erythrocytes take up sphingosines for S1P synthesis and release.

We show that erythrocytes can utilize exogenous sphingosines independently from Mfsd2b, for S1P synthesis. Exogenous sphingosine is quickly taken up in isolated erythrocytes and erythrocytes in the circulatory system. Intracellularly synthesized S1P is released into the blood stream in an Mfsd2b-dependent manner. We observed using fluorescent-labeled sphingosines (NBD-Sph and TMR-Sph) that sphingosine is quickly taken up by erythrocytes for S1P synthesis. S1P is mainly localized to the plasma membrane of erythrocytes. It is intriguing that we also observed the presence of vesicles-like structures in the cytoplasm of erythrocytes. Some of these fluorescence-labeled structures are found associated with the plasma membrane of erythrocytes. However, it is unclear how these structures are formed. It could be that S1P is bound to a cytosolic protein in erythrocytes as we did not observe the involvement of endocytosis of the sphingosine analogs in erythrocytes.

The efficient uptake capability of sphingosines in erythrocytes is uncharacterized. We noticed that deletion of Mfsd2b results in an approximately 10-fold increase of sphingosines, whereas lack of SphK1 only results in a twofold increase of these lipids (14, 19). Therefore, we hypothesized that the expression of SphK1 is necessary to increase the influx of sphingosines. Indeed, our results show that inhibition of SphK1 causes a reduction in S1P by reducing the influx of sphingosine into erythrocytes. The conversion of sphingosine to S1P by SphK1 thus enhances the uptake of sphingosine, likely by trapping the nondiffusable S1P form.

It is challenging to define the sources of circulating sphingosines for S1P synthesis in erythrocytes *in vivo*. We attempted to gain insight into this mechanism by taking advantage of the loss of S1P release activity in erythrocytes from Mfsd2b knockout mice. Thus, any changes in S1P levels in Mfsd2b KO erythrocytes would be attributed to S1P synthesis as release is blocked. We show that *de novo* synthesis of sphingolipids *via* SPT1 provides dihydro-sphingosine and indirectly provides other sphingosines (*e.g.*, Sph d18:1 and d18:2 species) for the synthesis of S1P species *in vivo*. In myriocin inhibition experiments, we observed a reduction of both dhS1P and other S1P species in erythrocytes. In contrast, treatment of washed erythrocytes with myriocin did not reduce S1P levels. These results indicate that *de novo* synthesis of sphingolipids in nonerythrocytes is involved in providing the source of circulating sphingosines. In line with this, sphingosines are detected in human and mouse plasma (19, 20, 31). In our conditions, myriocin treatment did not significantly reduce the total plasma S1P level, affecting only the dhS1P concentration. These observations may point to the possibilities that other pathways such as recycling sphingolipids also provide circulating sphingosines for S1P synthesis in erythrocytes. In line with this notion, alkaline ceramidase 2 was recently reported to provide dhSph and Sph for S1P synthesis in erythrocytes and platelets (32). However, it is unclear whether the hydrolysis of ceramides occurs in these anucleated cells or if other cells provide sphingosines for erythrocytes. The former scenario is unlikely as inhibition of alkaline ceramidase 2 does not result in increased ceramide levels in erythrocytes (32). Future investigations utilizing genetic inhibition of sphingolipid synthesis or degrading enzymes would be critical to define the source of sphingosines for S1P synthesis.

Recent cloning of Mfsd2b has opened up new avenues for understanding of how erythrocytes release S1P to the circulatory system. Mfsd2b is a conserved protein, which shares approximately 44% identity with the lysophosphatidylcholine transporter Mfsd2a (16, 25). Mfsd2a utilizes sodium to import lysophosphatidylcholine into the brain via the blood–brain barrier (16). In contrast, Mfsd2b does not use sodium and other cations or abundant anions, such as chloride, to drive the transport, even though the amino acid residues that are predicted to bind to sodium are conserved between Mfsd2a and Mfsd2b. Our mutagenesis results showed that D95 and T157 residues, which are, respectively, equivalent to the sodium-binding residues D97 and T159 in Mfsd2a, are required for Mfsd2b transport activity (14). The current results indicate that the carboxyl group in D95 and hydroxyl group in T157 are required for Mfsd2b activity, perhaps via interacting with proton during transport. The inhibitory effects of proton pump inhibitors to S1P export in erythrocytes further suggest that Mfsd2b utilizes a proton gradient to facilitate with S1P release. This characteristic of Mfsd2b resembles the transport mechanism of antiporters (33). Although a detail of the mechanism needs further investigations, it is possible that Mfsd2b behaves like an antiporter for proton/S1P.

The structure of several MFS transporters has been solved in recent years (17, 33). It shows that these transporters form a small cavity that allows the movement of ligands. In addition, they transport their ligands by switching protein conformations. To gain insights into whether the cavity of Mfsd2b can tolerate S1P analogs, we utilized NBD-Sph, fluorescein-Sph, and TMR-Sph as precursors to determine whether attachment of a fluorescent group to the omega carbon of the sphingoid backbone could interfere with the export of these S1P analogs. We show that NBD-S1P can be exported via Mfsd2b, suggesting that the attachment of the NBD group does not affect S1P release. However, Mfsd2b was unable to release TMR-S1P, indicating that the TMR group is unsuitable for transport via Mfsd2b. It remains to be determined whether the transport cavity of Mfsd2b is unable to tolerate the TMR group. Nevertheless, we find that TMR-S1P behaves like an “inside-out” inhibitor for Mfsd2b. Furthermore, TMR-S1P inhibits S1P release at lower concentrations, implying that it may interact with Mfsd2b. Indeed, our results from CETSA also support this possibility. S1P transporters may represent as new drug targets for treatment of diseases. For example, Spinster homolog protein 2 (Spns2) is an S1P transporter in lymphatic endothelial cells. Genetic inhibition of Spns2 results in lymphopenia, which protects the animals from various inflammatory conditions, highlighting its potential as a druggable target (34, 35). As Spns2 also belongs to MFS transporters, which might operate through a similar transport mechanism with Mfsd2b, we speculate that similar compounds like TMR-S1P may be developed as inhibitors for these S1P transporters.

In conclusion, our results emphasize that erythrocytes can utilize exogenous sphingosines from *de novo* synthesis and other unidentified cellular sources for S1P synthesis *in vivo*. Erythrocytes provide a substantial amount of plasma S1P via Mfsd2b. We reveal that Mfsd2b prefers a proton gradient to maximize its transport activity. Our results implicate that proton pump targeting drugs might affect S1P release from erythrocytes. Finally, we show that TMR-S1P can function as an inhibitor of Mfsd2b in erythrocytes.

## Experimental procedures

### Mice

Whole body knockout mice of Mfsd2b were generated as described (14). All mice were in C57BL/6 background. Erythrocytes-specific knockout Mfsd2b mice were generated by intercrossing homozygous floxed Mfsd2b (Mfsd2bf/f) mice with Mfsd2bf/f mice containing a copy of EpoR-Cre (36). RBC-specific knockout EpoR-GFP-Cre mice was genotyped by the forward primer GTGTGGCTGCCCCTTCTG and the reverse primer CAGGAATTCAAGCTCAACCTCA (36). Deletion of Mfsd2b in RBC was confirmed by Western blot and S1P transport activity. Global Spns2 knockout mice were obtained from KOMP. Mice were maintained on normal chow diets. All experimental protocols were approved by institutional animal care and use committees under National University of Singapore.

### Chemicals

Sphingosine (Sph, D-erythro-sphingosine) and sphingosine-1-phosphate (D-erythro-sphingosine-1-phosphate) were purchased from Avanti. Radiolabeled [3-^3^H]sphingosine ([3-^3^H]-Sph) (Stock specific activity: 1 μCi/μl) was purchased from American Radiochemicals. Fatty acid–free BSA was purchased from Sigma. All other reagents were purchased from Sigma. NBD-sphingosine (NBD-sph) (omega(7-nitro-2-1,3-benzoxadiazol-4-yl)(2S,3R,4E)-2-aminooctadec-4-ene-1,3-diol) was purchased from Avanti Lipids. Fluorescein-sphingosine (Catalog, S100F) and tetramethylrhodamine-sphingosine (TMR-Sph) (Catalog, S100T) were purchased from Echelon Biosciences. These sphingosine analogs were solubilized in 12% BSA in 150 mM NaCl or in ethanol.

### Antibody

Rabbit polyclonal antibodies for Mfsd2b were generated as described (14) and used at 1:500 for Western blot. The Spns2 antibody was generated using 22 amino acids from the C terminus of human Spns2 protein (SDRARAEQQVNQLAMPPASVKV) and validated with Spns2 protein overexpression in HEK293 cells. Western blot membranes were reprobed with Gapdh or actin antibody for loading control.

### Mutagenesis

To generate the mutant plasmids for aspartate 95 and threonine 157 residues, we used the PCR mutagenesis method. This method has been published elsewhere in the literature (14).

### Collection of plasma and RBC for lipidomics analysis

For plasma collection, peripheral blood was collected in heparinized capillaries into EDTA-K2 tubes. Plasma was separated from blood cells by centrifugation at 2500 rpm at room temperature for 30 min. For RBC preparation, blood was collected in EDTA-K2 tubes and washed thoroughly at room temperature by Tyrode H buffer (10 mM Hepes-NaOH,12 mM NaHCO3, 138 mM NaCl, 5.5 mM D-glucose, 2.9 mM KCl, 1 mM MgCl2, pH 7.4) and the number of RBCs was numerated with a hematology analyzer Celltac Alpha MEK-6500K (Nihon Kohden). Lipidomics was performed using liquid chromatography/mass spectrometry (LC/MS). For measurements of sphingosine 1-phosphate, RBC and plasma samples were first spiked with internal standards and subjected to lipid extractions using a 1-butanol/methanol (1:1) mixture. The level of individual S1P was quantified and normalized to internal standards as described (14, 37) and expressed as relative levels indicated in the figures.

### Transport assays

We utilized two slightly different transport assays. In “continuous” assays, 1 to 2.5 μM [3-^3^H]-Sph dissolved in 12% BSA (1 mM stock) was incubated with 2 × 10^7^ erythrocytes for 30 to 60 min in Tyrode-H buffer containing 0.5% BSA. The supernatant and cell pellets were collected for S1P isolation. In “preloading” assays, 1 to 2.5 μM [3-^3^H]-Sph (1 mM stock) substrate dissolved in ethanol was preincubated with erythrocytes in Tyrode-H buffer without BSA (thus, there is no release of S1P) for 30 to 60 min. Cells were washed once with Tyrode-H buffer to remove the radioactive substrate and then incubated for 30 to 60 min in Tyrode-H buffer containing 0.5% BSA to stimulate S1P release. In both assays, the supernatant and cell pellets were collected for S1P isolation and scintillation counting. The specific activity of [3-^3^H]-Sph in 1 mM Sph containing 12% BSA was 10 μCi/ml. For drug treatments, 10 μM bafilomycin A1 (BA1), 100 μM omeprazole (OPZ), or 200 μM glyburide was used. For CCCP treatment, 0.5 or 1 mM CCCP was used.

### Measurement of S1P release using thin layer chromatography

Washed RBCs were prepared and resuspended at a density of 2 × 10^7^ erythrocytes in Tyrode-H buffer (pH 7.4) and incubated with 2.5 μM NBD-Sph or TMR-Sph for 1 h at 37 °C. Lipids from the supernatant and cell pellets were isolated. Lipids were extracted using organic solvents and spotted on precoated silica plates. TLC plates were run with solvent containing 3 volumes of 1-butanol, 1 volume of acetic acid, and 1 volume of water. The TLC plates were dried and visualized by GelDoc (Bio-Rad), and the fluorescence signal was quantified by Image Lab software (Bio-Rad). NBD-Sph, fluorescein-Sph, or TMR-Sph was used as standards in TLC analysis.

### Competition assays with NBD-Sph, fluorescein-Sph, or TMR-Sph

The competition assays with fluorescent sphingosines were similarly performed as described above for S1P transport assays with slight modifications. Briefly, 2 × 10^7^ erythrocytes from WT and Mfsd2b KO mice were preincubated with 2 μM fluorescent sphingosines as indicated in the figure legends for 2 h, then 2 μM [3-^3^H]-Sph and 0.5% BSA were added. The mixture was further incubated for 30 min. Then, the supernatant and cell pellets were collected for S1P isolation and scintillation counting.

### Time course and dose curve of TMR-Sph

For these assay, 2 × 10^7^ erythrocytes from WT and Mfsd2b KO mice were preincubated with either TMR-Sph or unlabeled sphingosine. For time course, the cells were preincubated with 2 μM of TMR-Sph or unlabeled sphingosine for 10, 30, 60, and 120 min. For the dose curve, the cells were preincubated for 0.5, 1, 2, or 4 μM TMR-Sph or equal molars of unlabeled sphingosine for 2 h. After the preincubation, the mixtures of both assays were supplemented with 1 μM [3-^3^H]-Sph and 0.5% BSA. The mixture was further incubated for 30 min before the supernatant and cell pellets were collected for S1P isolation and scintillation counts.

### *In vivo* transport assays with [3-^3^H]-sphingosine

Mice were injected with 1 μl/g body weight from 1 mM [3-^3^H] sphingosine solubilized in 12% BSA (specific activity: 10 μCi/ml). An equal small amount of blood was collected in EDTA tubes. Blood cells were spun down and the plasma was collected. To harvest the remaining plasma in the first collection, the cell pellets were washed once with Tyrode-H containing 0.1% BSA and spun down to collect the supernatant. This supernatant was combined with the plasma sample in the first collection. The cell pellets and plasma samples were kept for S1P isolation. Experiments were performed for 10, 30, 60, 120, and 180 min. The radioactive signals from the S1P fractions were normalized to that of WT at 10 min.

### PF543 inhibition assays

Blood was collected into EDTA-K2 tubes as described above. Erythrocytes were washed twice with Tyrode-H buffer and numerated. Amounts of 2 × 10^7^ erythrocytes were used in transport assays with or without PF543. Erythrocytes were pretreated with 20 μM PF543 or an appropriate volume of dimethyl sulfoxide (vehicle) in Tyrode-H buffer for 15 min. The cells were spun down at 2500 rpm for 5 min using a Beckman Swinging rotor to remove the buffer. Erythrocytes were subsequently treated with 20 μM PF543 or 60 μM PF543 in Tyrode-H buffer containing 0.5% BSA and 2 μM [3-^3^H] sphingosine for 15 min. The cell pellets and supernatant were subjected to S1P extraction for scintillation counts.

### Super resolution microscopy

Blood was collected into tubes containing 5% EDTA-K2 and washed immediately twice using Tyrode-H buffer. Erythrocytes were counted using the Celltac α hematology analyzer (Nikon Kohden). A total of 2 × 10^7^ erythrocytes were incubated with 2.5 μM NBD-Sph (Avanti Lipids) or TMR-Sph (Echelon Biosciences) in 200 μl of Tyrode-H buffer supplemented with 0.5% BSA at 37 °C. RBCs were collected at 2 to 3 different time points, 1, 5, and 30 min, by immediately fixing with 1% paraformaldehyde. RBCs were then imaged under a super resolution microscope (Nikon eclipse Ti).

### Drug treatment

Myriocin was intraperitoneally injected into WT and KO mice at a dose of 0.3 mg/kg every 2 days for 24 days. Erythrocytes and plasma samples were collected from untreated mice and myriocin-treated mice after 24 days as described above. For myriocin or amitriptyline treatment with isolated erythrocytes, 500 million RBCs were incubated with 10 μM myriocin for 4 h or 100 μM amitriptyline for 24 h in Tyrode-H buffer without BSA. The cells were washed once and collected for lipidomics.

### Cellular thermal shift assay

Washed RBCs were preincubated with 2 μM TMR-Sph or vehicle in Tyrode-H buffer for 1 h. The excess amount of TMR-Sph was removed by centrifugation. The same amount of TMR-Sph-treated cells was heated in a PCR machine at different temperatures for 15 min. Precipitates were removed by centrifugation, and the same amount of supernatant was used for Western blot analysis. The intensity of the Mfsd2b protein band incubated with TMR-Sph was quantified and normalized to the Mfsd2b protein band incubated with the vehicle at each temperature.

### Western blot analysis

Tyrode-H-washed erythrocytes were lysed by RIPA buffer (25 mM Tris with pH 8.0, 150 mM NaCl, 0.1% SDS, 0.5% sodium deoxycholate 0.5% Triton X-100) supplemented with protease inhibitor cocktail (Roche). Protein lysates of 100 to 150 μg were prepared in Laemmli loading buffer, resolved on 10% SDS-PAGE, and transferred to a nitrocellulose membrane. A LiCOR-IR-based system was used to detect and quantify the proteins of interest.

### Sphingomyelinase assay

The activity of sphingomyelinase of WT and Mfsd2b KO RBCs was measured using SMase activity assay (Catalog #K574-100, BioVision). In brief, 200 million RBCs from WT and Mfsd2b KO mice were lysed in a 50 μl mixture solution of SMase assay buffer and SMase extraction buffer for 15 min on ice. Subsequently, 1 μl of RBC lysates was diluted with 24 μl SMase assay buffer and then mixed with reaction reagents following manufacture's instruction. The SMase activity assay was performed in 96-well clear plates with flat bottom (Corning) and run in kinetic mode for 60 min. The fluorescent signals were measured at excitation 535 nm and emission 587 nm using a SpectraMaxM2 microplate reader (Molecular Devices, LLC).

### Statistical analysis

Data were analyzed using GraphPrism7 software for Windows. Statistical significance was calculated using *t* test or one and two-way ANOVA. *p* Value < 0.05 was considered as statistically significant.

## Data availability

Original data are available upon request to Long N. Nguyen, National University of Singapore. bchnnl@nus.edu.sg.

## Conflict of interest

The authors declare that they have no conflicts of interest with the contents of this article.
