# Supplementary material for: Erythrocytes efficiently utilize exogenous sphingosines for S1P synthesis and export via Mfsd2b
Source: J Biol Chem. 2021 Jan 23;296:100201. doi: 10.1074/jbc.RA120.012941 (PMC7948482; doi:10.1074/jbc.RA120.012941)
Supplement: Figures S1 to S6 [file mmc1.pdf]

Erythrocytes efficiently utilize exogenous sphingosines for S1P synthesis and export via Mfsd2b

*Toan Q Nguyen[1], *Thiet Minh Vu[1,6], *Farhana Tukijan[1], Sneha Muralidharan[2], Juat Chin Foo[1], Jasmine Fei Li Chin[3], Zafrul Hasan[1], Federico Torta[1], #Long N Nguyen[1,4,5]

Running title: Mechanisms of sphingosine-1-phosphate transport by Mfsd2b.

Affiliations:

[1]Department of Biochemistry, Yong Loo Lin School of Medicine, National University of Singapore.

[2]Department of Medicine, Yong Loo Lin School of Medicine, National University of Singapore.

[3]Mechanobiology Institute, National University of Singapore, Singapore 117411.

[4]SLING/Immunology Program, Life Sciences Institute, National University of Singapore, Singapore 117456.

[5]Immunology Translational and Cardiovascular Disease Research Programme, Yong Loo Lin School of Medicine, National University of Singapore, Singapore 117545.

[6]Current address: NTT Hi-Tech Institute, Nguyen Tat Thanh University, Ho Chi Minh City, Viet Nam.

*These authors contribute equally.

#Corresponding author (bchnnl@nus.edu.sg).

Key words: Sphingosine, Sphingosine-1-phosphate, S1P transporter, Mfsd2b.

Supporting information.

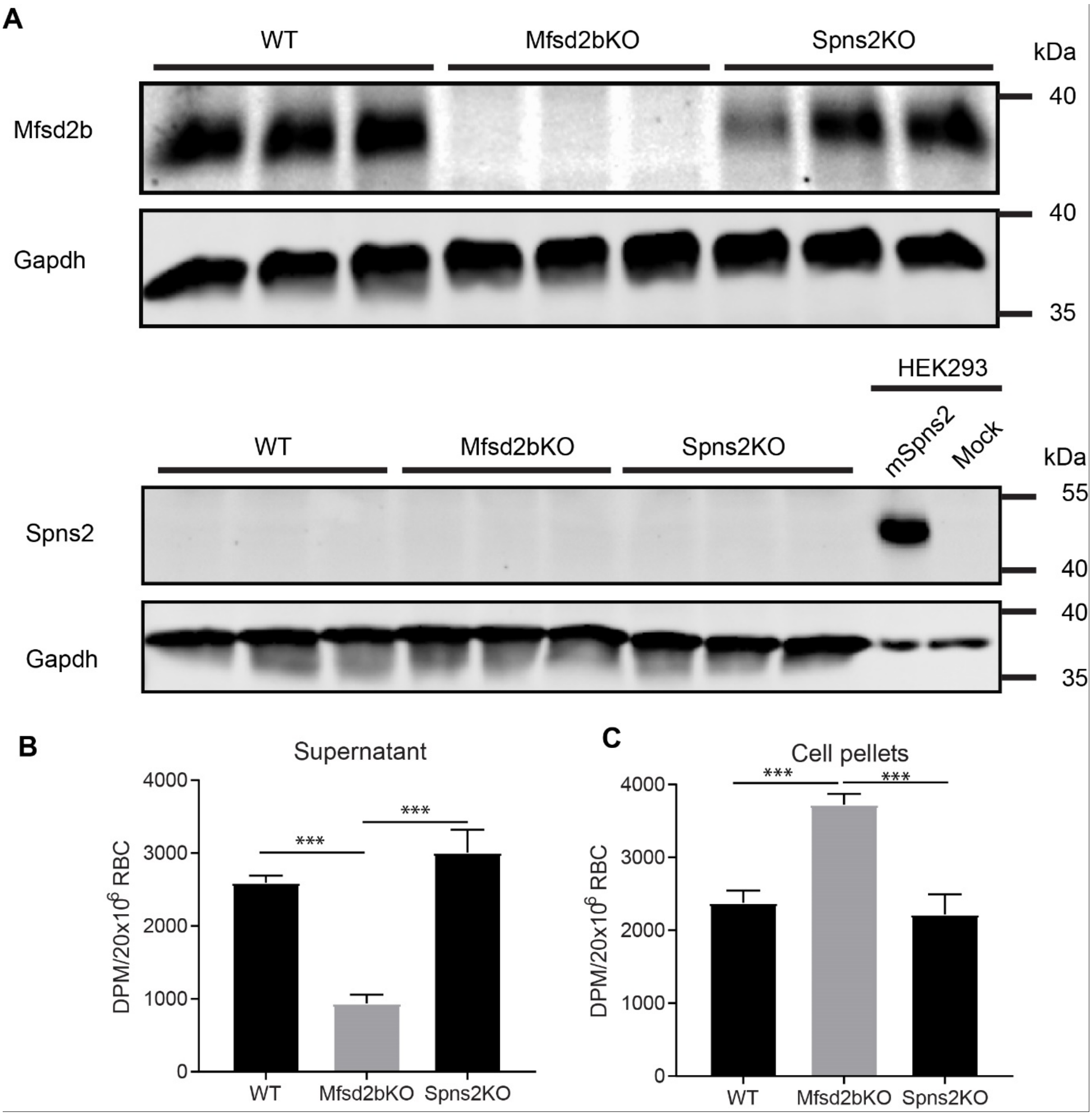

Supplemental figure 1. Erythrocytes export S1P via Mfsd2b, but not Spns2. *A, Western blot analysis of Mfsd2b and Spns2 in erythrocytes (RBC) isolated from wild-type (WT), global Mfsd2b KO, and global Spns2 KO mice, respectively. Shown are representative images from at least 3 experiments. Murine Spns2 protein from HEK293 was used to validate Spns2 antibody.* ***B,*** *Extracellular S1P levels from transport assays using RBC isolated from WT, Mfsd2b, and Spns2 knockout mice.* ***C****, Intracellular S1P levels from transport assays using RBC isolated from WT, Mfsd2b KO, and Spns2 KO mice. 2.5µM [3-^{3}H]-Sph was used in "continuous" assays for 30mins. The data show that Mfsd2b is the transporter that is responsible for S1P release in erythrocytes. Experiments were repeated at least twice. Data are mean and SD, n=3-4 per genotypes. ***P<0.001. P values were calculated using one-way ANOVA. DPM, disintegrations of radioactive per minute.*

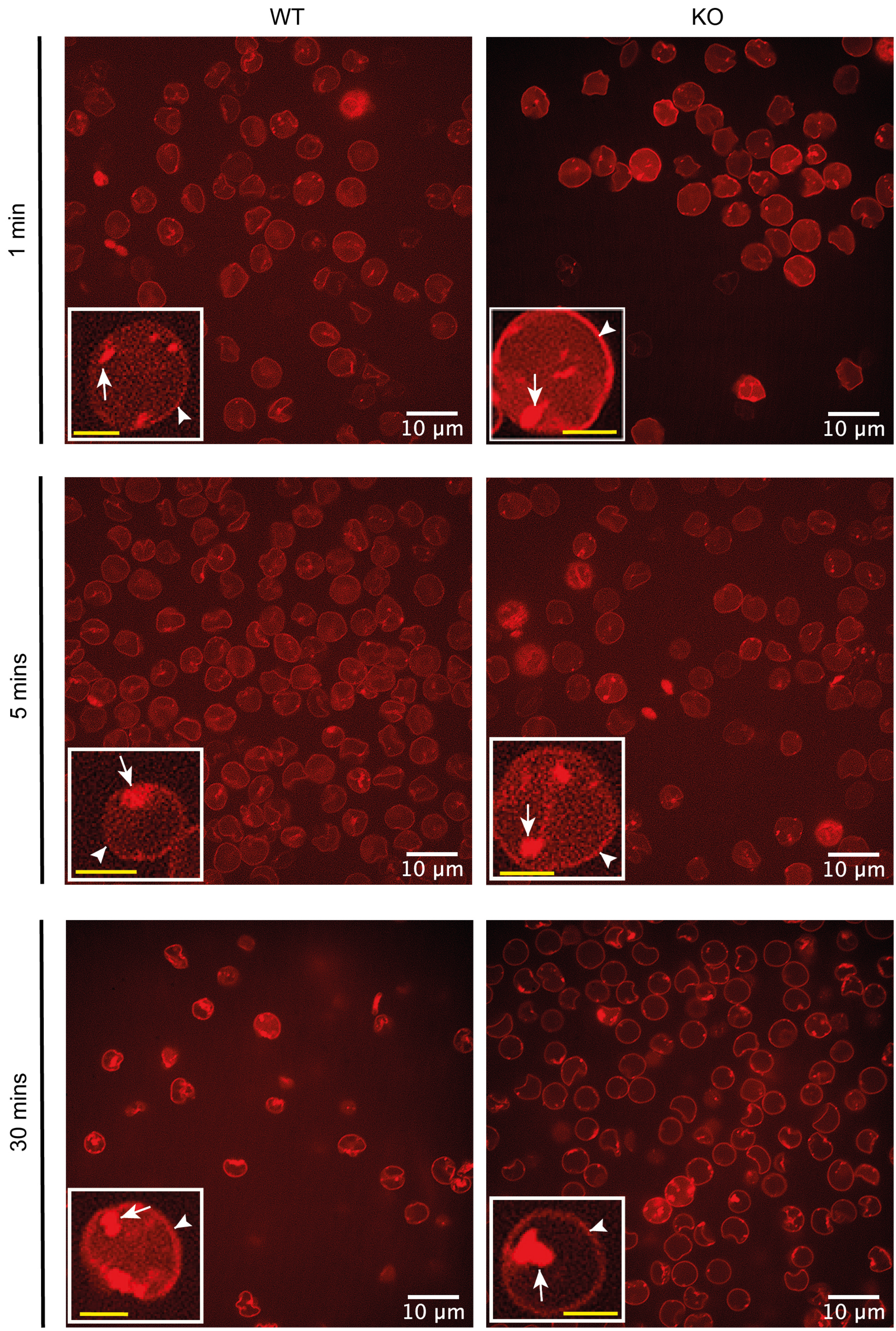

WT
KO
1 min
5 mins
30 mins
10 μm
10 μm
10 μm
10 μm
10 μm
10 μm

Supplemental figure 2. **Localization of TMR-S1P in WT and Mfsd2b KO RBC**. *After incubation with TMR-Sph for 1, 5 and 30 min(s), cells were fixed by 1% paraformaldehyde and then imaged under super resolution microscope. The arrows show the localization of TMR-Sph or TMR-S1P present in the cytoplasm. The arrow heads show the TMR signal in the plasma membrane. Representative images from 3 WT and 3 KO mice. Scale bar in insets: 2.5μm.*

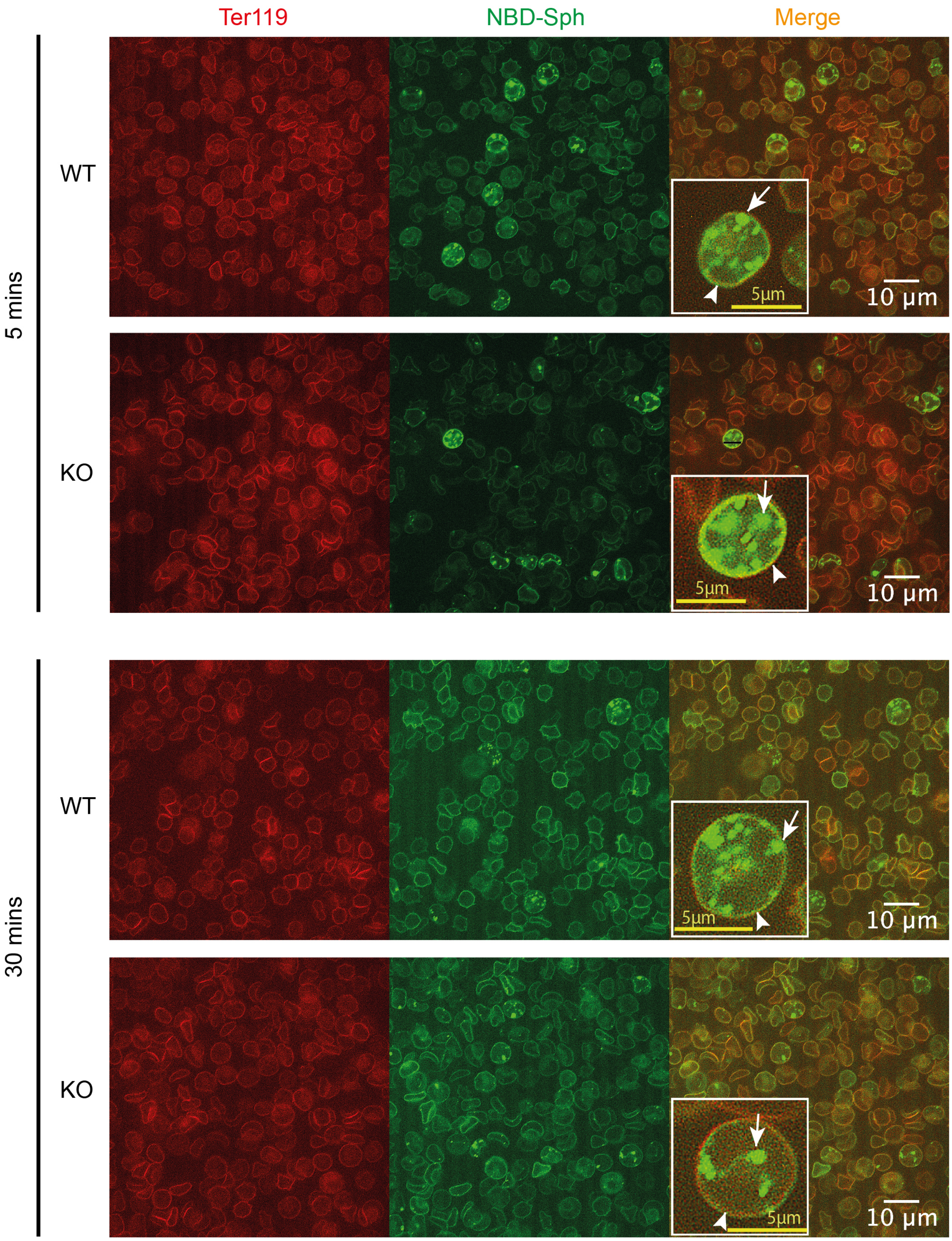

Supplemental figure 3. **Uptake of NBD-Sph in RBC is not involved in endocytosis.** ***The*** *plasma membrane of WT and Mfsd2b KO RBC were pre-labeled with Ter119 before incubating with NBD-Sph. The import of NBD-Sph was stopped at 5 and 30 mins prior to imaging of NBD (in green) and Ter119 (in red) signal. Note that NBD positive structures were present. However, they are not co-localized with Ter119, ruling out the endocytosis of NBD-Sph. Shown are representative images from 3 WT and 3 KO.*

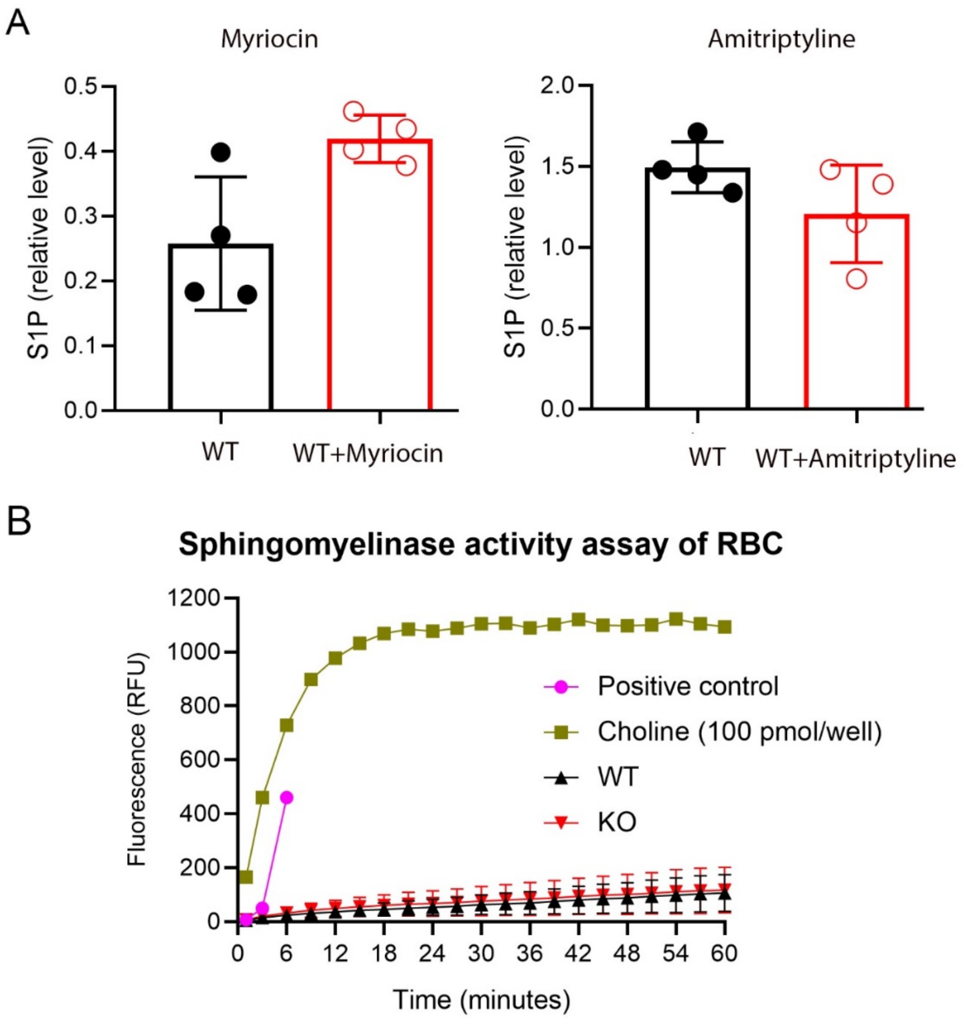

Supplemental figure 4. De novo synthesis of sphingolipids and hydrolysis of sphingomyelins are not active in erythrocytes. ***A***, *Myriocin and amitriptyline treatment do not reduce S1P level in wash RBC.* ***B***, *Neutral sphingomyelinase (SMase) activity of RBC was measured with a sphingomyelinase fluorometric assay kit following the manufacture's instruction. Compared with choline standard and the provided positive control, sphingomyelinase activity was not detectable from WT and KO RBC. Note that SMase activity of positive control (circle) got oversaturated after 12 mins. Data are mean and SD, n=4 per genotype.*

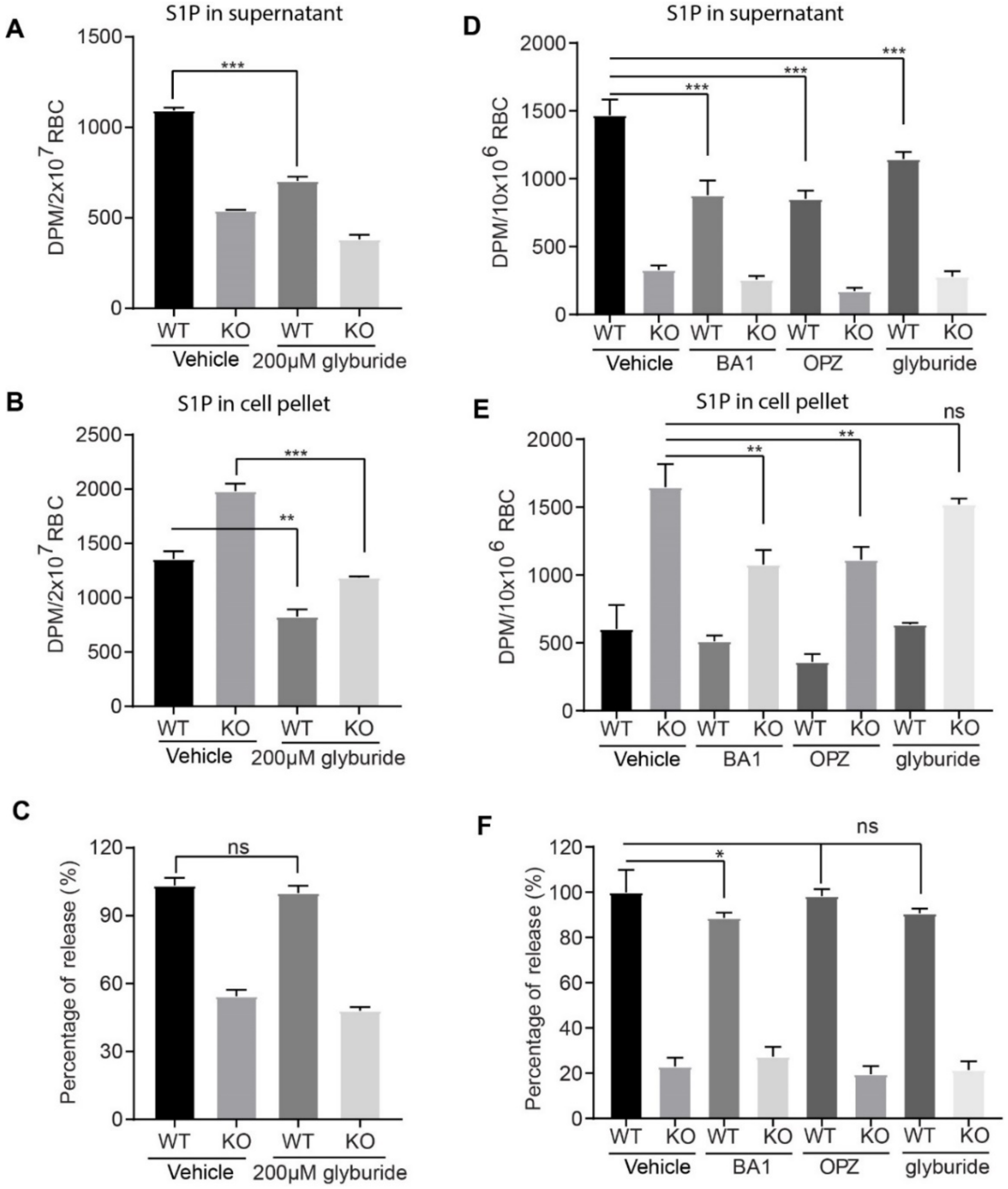

Supplemental figure 5. Proton pump inhibitors affect S1P synthesis and release in erythrocytes. ***A-C****, Effects of glyburide on the transport of S1P in erythrocytes. Erythrocytes were pre-treated with glyburide. Radioactive [3-^{3}H]-sphingosine was added in "continuous transport" assays for 30mins. S1P in the supernatant and cell pellets were isolated and quantified. Results showed that glyburide reduced total S1P amount for maximum release rather than directly inhibiting export.* ***D-F****, WT and KO RBC were pretreated with 10µM BA1, 100µM OPZ and 200 µM glyburide for 30 mins. Then, [3-^{3}H]-sphingosine was incubated with the cells to preload S1P in "preloading" assay before addition of 0.5% BSA to stimulate S1P release. WT RBC under indicated drug treatments showed lower S1P release compared to that of vehicle treatment. The reduced release of S1P in WT cells did not significantly result in increases in intracellular S1P level, indicative of a reduction in total amount of S1P. Thus, the percentage of S1P release in WT cells under drug treatments and vehicle were comparable. Data are means and SD. Data are expressed as mean and SD, n=3 per genotype. Experiments were repeated twice. ***P<0.001, ** P<0.01; ns: not significant. P values were calculated using one-way ANOVA.*

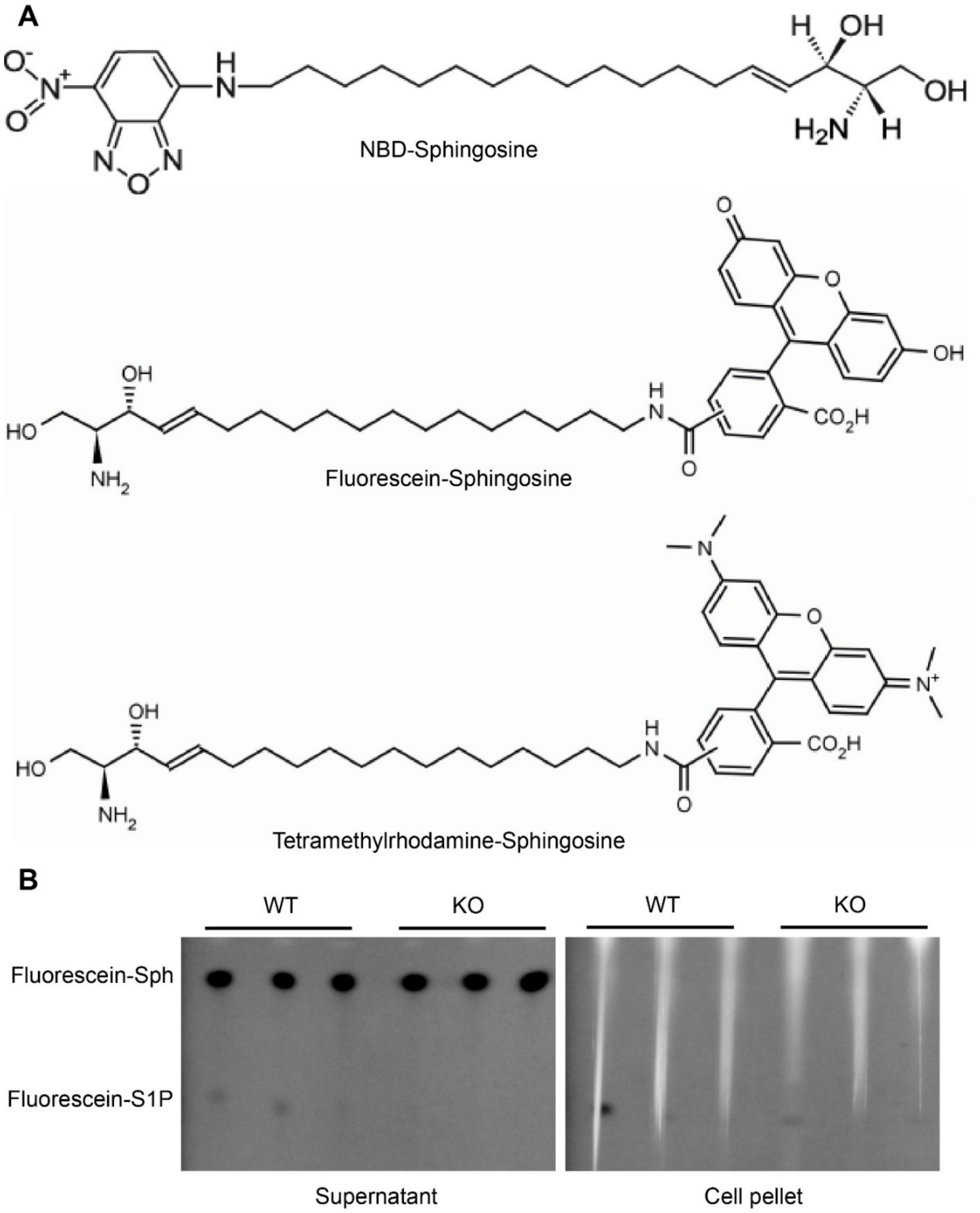

Supplemental figure 6. **Sphingosine analogs**. *A, structures of sphingosine analogs that used in competition assays.* ***B****, Thin layer chromatography analysis of fluorescein-S1P after WT and Mfsd2b knockout erythrocytes were incubated with 2.5 µM fluorescein-sphingosine in "continuous" assays. Note that unlike NDB-sphingosine and TMR-sphingosine, fluorescein-sphingosine is not efficiently taken up by erythrocytes. Thus, fluorescein-sphingosine was remained in transport buffer.*
